# N-acetyl-L-cysteine Improves the Developmental Competence of Bovine Oocytes and Embryos Cultured In Vitro by Attenuating Oxidative Damage and Apoptosis

**DOI:** 10.3390/antiox10060860

**Published:** 2021-05-27

**Authors:** Wu-Sheng Sun, Hoon Jang, Mi-Ryung Park, Keon Bong Oh, Haesun Lee, Seongsoo Hwang, Li-Jie Xu, In-Sul Hwang, Jeong-Woong Lee

**Affiliations:** 1 Animal Biotechnology Division, National Institute of Animal Science, Rural Development Administration, Jeollabuk-do 55365, Korea; sunwusheng@163.com (W.-S.S.); mrpark45@korea.kr (M.-R.P.); keonoh@korea.kr (K.B.O.); leehs1498@korea.kr (H.L.); hwangss@korea.kr (S.H.); 2 Biotherapeutics Translational Research Center, Korea Research Institute of Bioscience and Biotechnology, Daejeon 34141, Korea; 3 Department of Life Science, Jeonbuk National University, Jeollabuk-do 54896, Korea; hoonj@jbnu.ac.kr; 4 Guangdong AIB Polytechnic College, Guangzhou 510507, China; dsd0765@126.com

**Keywords:** NAC, oocyte, mitochondria, ROS, apoptosis, in vitro maturation

## Abstract

Oxidative stress has been suggested to negatively affect oocyte and embryo quality and developmental competence, resulting in failure to reach full term. In this study, we investigated the effect of N-acetyl-L-cysteine (NAC), a cell-permeating antioxidant, on developmental competence and the quality of oocytes and embryos upon supplementation (0.1–10 mM) in maturation and culture medium in vitro using slaughterhouse-derived oocytes and embryos. The results show that treating oocytes with 1.0 mM NAC for 8 h during in vitro maturation attenuated the intracellular reactive oxygen species (ROS) (*p* < 0.05) and upregulated intracellular glutathione levels (*p* < 0.01) in oocytes. Interestingly, we found that NAC affects early embryonic development, not only in a dose-dependent, but also in a stage-specific, manner. Significantly (*p* < 0.05) decreased cleavage rates (90.25% vs. 81.46%) were observed during the early stage (days 0–2), while significantly (*p* < 0.05) increased developmental rates (38.20% vs. 44.46%) were observed during the later stage (from day 3) of embryonic development. In particular, NAC supplementation decreased the proportion of apoptotic blastomeres significantly (*p* < 0.05), resulting in enhanced hatching capability and developmental rates during the in vitro culture of embryos. Taken together, our results suggest that NAC supplementation has beneficial effects on bovine oocytes and embryos through the prevention of apoptosis and the elimination of oxygen free radicals during maturation and culture in vitro.

## 1. Introduction

Slaughterhouse-derived oocytes are typically used for the large-scale production of in vitro embryos in many livestock. However, when ovaries are transported over long distances from the slaughterhouse to the laboratory, the lack of oxygen and energy supply and disorders of the endogenous antioxidant system in isolated ovaries may affect the quality of the oocytes in the follicles [1]. Furthermore, the quality of oocytes and embryos cultured in vitro is still not as high as that of in vivo, although great progress has been made in optimizing in vitro culture systems [2]. There are many reasons for these differences, one of which is oxidative stress (OS); reactive oxygen species (ROS) are prominent mediators associated with oocyte damage and cause poor embryonic development [3,4].

In order to reduce these anomalously generated ROS in oocyte and embryos, various antioxidants have been used in vitro cultures [5]. The major cellular antioxidants are enzymatic substances, such as superoxide dismutase (*SOD*), catalase (*CAT*), and Se-dependent glutathione peroxidase (*GPx*), and non-enzymatic low molecular substances, such as reduced glutathione (GSH) [6]. GSH is important for the elimination of a variety of toxic substances, including xenobiotics and peroxide, and performs an essential function in cell protection [7]. However, oocytes and early embryos that have developed to the stage before the blastocyst stage can only synthesize a limited amount of GSH, and GSH outside the cell cannot penetrate the cell membrane and enter the cells. Therefore, their acquisition of GSH at this stage depends on the cumulus cells absorbing thiols, which then synthesize GSH through the γ-glutamyl cycle [8]. Supplementing culture media with thiol compounds, such as L-cysteine, has been shown to improve the maturation rate of oocytes [9] and increase the developmental potential of early embryos [10,11,12]. However, the bioavailability of L-cysteine is low, as it is easily oxidized to L-cystine and is depleted within a few hours [13]. Its concentration can be reduced to 40% and 3% of its initial concentration after 3 h and 24 h of in vitro maturation (IVM) medium of bovine oocytes [14].

N-acetyl-L-cysteine (NAC), a derivative of L-cysteine, can overcome the problem of L-cysteine’s low bioavailability by being inserted into an acetyl group to extend its half-life [15]. The molecular structure of NAC allows it to easily penetrate the cell membrane directly and then be deacetylated to L-cysteine, which can be combined with glutamate and glycine to produce GSH inside the cells [16]. NAC has multiple biological activities such as immune regulation, anti-apoptosis, and antioxidation [17,18,19,20]. Within the range of millimolar use, NAC is reported to be more efficient in removing HOCl than L-methionine, α-lipoic acid and L-cysteine [21]. Moreover, results from animal studies have confirmed that NAC is neither teratogenic nor mutagenic [22] and can be safely used in humans [23]. Previous studies showed that treatment with 1.0 mM NAC in vivo could delay oocyte aging in mice [24,25] and protect porcine oocytes against the oxidative stress and apoptosis induced by heat stress [26], as well as increase *SOD* activity by reducing the degree of DNA fragmentation [27]. However, up until now, there have been limited studies regarding the beneficial effects of NAC on bovine oocyte and embryo development, except for one study, which demonstrated that the addition of 0.6 mM NAC did not significantly improve the quality of bovine oocytes and embryos; the authors inferred that the reason may be because NAC inhibits NF-κB pathway activation during oocyte maturation [28]. Moreover, they did not study the dose effect and stage-specific effects of NAC on oocyte maturation and embryo development. Hence, we propose that more studies need to be performed. In the present study, three concentrations (0.1 mM, 1.0 mM and 10 mM) and six restricted time windows (8 h, 16 h and 22 h in IVM medium; 0–3 dpf, 3–6 dpf and 6–9 dpf in IVC medium) for NAC treatment were chosen to systematically evaluate the effects of NAC on bovine oocytes and embryos. In addition, 0.6 mM L-cysteine, a commonly used concentration in oocyte IVM, was selected as a reference. This study will enhance our understanding of the role of NAC in bovine oocyte maturation and embryo development.

## 2. Materials and Methods

### 2.1. Chemicals

All the chemicals used in this study were purchased from Sigma Aldrich (St. Louis, MO, USA), unless otherwise stated.

### 2.2. Oocyte Collection and In Vitro Maturation

Ovaries were obtained from a local slaughterhouse and transported to the laboratory in a 0.9% salt solution at 37 °C within around 6~8 h after slaughter. The follicular fluid was aspirated from antral follicles (3–6 mm in diameter) with an 18-G needle connected to a 10 mL syringe. Cumulus oocyte complexes (COCs) were then collected under a stereo microscope and cultured in Medium 199 (Life Technologies Corporation, Grand Island, NY, USA) containing 10% (*v*/*v*) fetal bovine serum (FBS, Life Technologies Corporation, Grand Island, NY, USA), 10 ng/mL epidermal growth factor, 10 μg/mL follicle-stimulating hormone, 1.0 μg/mL estradiol, 0.2 mM sodium pyruvate and 1% penicillin/streptomycin sulfate solution (Life Technologies Corporation, Grand Island, NY, USA) (IVM medium) for 22 h at 39 °C in 5% CO_2_.

### 2.3. In Vitro Fertilization (IVF) and In Vitro Culture (IVC)

IVF was performed as described previously [29]. Briefly, a 0.5-mL straw of Hanwoo semen was thawed at 37 °C for 30 s; then, the contents in the straw were layered onto the top of a Percoll density gradient consisting of 2 mL of 45% Percoll above 2 mL of 90% Percoll in a 15 mL conical tube. After being centrifuged at 700× *g* for 20 min, the sperm pellet was washed twice in 4 mL of modified Brackett and Oliphant (mBO) medium (IVF100, Research Institute for the Functional Peptides, Tokyo, Japan), supplemented with 5 mM theophylline at 300× *g* for 5 min each time. Finally, the sperm pellet was resuspended in mBO medium supplemented with 5 mg/mL bovine serum albumin (BSA), 10 μg/mL heparin and 1.0 mM caffeine (IVF medium) to yield a concentration of 2 × 10^6^ spermatozoa/mL. Then, 15 COCs were co-incubated with spermatozoa in a 100 μL IVF medium microdrop for 6 h at 38.5 °C under a humidified atmosphere in 5% CO_2_. After IVF, 10–15 presumptive zygotes were cultured in a 55 μL microdrop of CR1aa medium containing 114.7 mM NaCl, 31.0 mM KCl, 26.2 mM NaHCO_3_, 0.4 mM sodium pyruvate, 0.01 g/L phenol red, 1.0 mM glutamine, 2.75 mM calcium L-lactate, 1% (*v*/*v*) MEM non-essential amino acids, 1% (*v*/*v*) BME essential amino acids, 0.3% (*w*/*v*) BSA and 1% (*v*/*v*) penicillin/streptomycin (Life Technologies Corporation, Grand Island, NY, USA) at 38.5 °C for 2 days in 5% CO_2_. The culture medium was changed every 3 days and replaced with fresh CR1aa culture medium supplemented with 5% (*v*/*v*) FBS. The cleavage rate was determined at 2 days post fertilization (2 dpf), and the blastocyst rate was recorded on 7–8 dpf and calculated as total blastocysts to cleaved oocytes.

### 2.4. Total RNA Extraction and Real-Time RT-PCR

Total RNA was isolated from cumulus cells or ZP-free oocytes and embryos using the Trizol reagent (Invitrogen), according to the manufacturer’s protocol. The cDNA was synthesized using the TOPscript Reverse Transcription kit (Enzynomics Co. Ltd., Daejeon, Korea). The mRNA levels of *NFKB1*, *Bax*, *Bcl-2*, *CAT*, and *SOD*1 genes were detected by real-time PCR. The reaction was performed as follows: 45 cycles of 15 s for denaturation at 95 °C, 15 s annealing at 60 °C, 20 s extension at 72 °C and a melting curve analysis at the end of the run. Information on the primers is shown in Table 1. The relative expression of *GAPDH* was used as an internal control.

### 2.5. Measurement of Intracellular ROS, GSH Levels and Mitochondrial Membrane Potential

The intracellular ROS content was measured according to our previous study [29]. In brief, denuded oocytes were incubated in DPBS containing 0.4% (*w*/*v*) polyvinyl alcohol (DPBS-PVA) and 10 μM 2′,7′-dichlorofluorescein diacetate (H_2_DCFDA) at 38.5 °C in 5% CO_2_ for 30 min in the dark. Oocytes were washed three times in DPBS-PVA, and the fluorescence signal from each oocyte was analyzed under an Olympus IX83 inverted fluorescence microscope (Olympus, Tokyo, Japan). The level of GSH in each oocyte was measured with 10 μM 4-chloromethyl-6.8-difluoro-7-hydroxycoumarin (Cell-Tracker Blue, Invitrogen, CA, USA). The experimental procedure was the same as the ROS measurement described above. The mitochondrial membrane potential was measured with a JC-1 mitochondrial membrane potential assay kit (Cayman Chemical, MI, USA) according to the manufacturer’s protocol. The fluorescence intensity in the captured images was quantified by using the ImageJ software.

### 2.6. Cell Proliferation Assay

Cell viability was determined with a WST-based cell viability assay kit (Dogen, Korea) according to the manufacturer’s instructions. Briefly, COCs were denuded with 1 mg/mL hyaluronidase and cumulus cells were collected and plated onto a 96-well plate at 5 × 10^4^ cells/well in 100 μL of Dulbecco’s Modified Eagle Medium (Welgene, Korea) supplemented with 10% FBS. Next, 10 μL reaction buffer was added to each well and incubated for 4 h at 38.5 °C in 5% CO_2_. Then, colorimetric measurements were performed with a plate reader (BioTek Gen 5, VT, USA) at 450 nm. The experiments were performed in triplicate. Cell viability was calculated as follows: relative viability (%) = [A_450 (treated)_ − A_450 (blank)_]/[A_450 (control)_ − A_450 (blank)_] × 100% [30].

### 2.7. Total Antioxidant Capability Measurement

The total antioxidant capability (TAC) levels of cumulus cells were measured according to a previous study [31], with some modifications, using a Total Antioxidant Capability Assay kit (Dogen, Korea). Briefly, COCs were denuded and 5 × 10^5^ cumulus cells in different treatment groups were homogenized in 100 μL Dulbecco’s phosphate-buffered saline (DPBS) and sonicated, before being centrifuged at 3000× *g* for 5 min at 4 °C. The cellular supernatant was added to the Copper Reagent in a 96-well microplate and incubated at room temperature for 30 min. The absorbance was measured in a microplate reader at 450 nm.

### 2.8. Apoptosis Assays and Total Blastocyst Cell Counting

Cell apoptosis was assessed by an In Situ Cell Death Detection kit (Roche, Monza, Italy). For the cumulus cell assay, the cells were seeded onto coverslips on a 12-well culture plate 1 day before staining. For embryo staining, blastocysts were harvested on 8 dpf. Then, the samples were washed 3 times with DPBS-PVA and fixed in DPBS-PVA containing 4% (*w*/*v*) paraformaldehyde at room temperature for 30 min. The fixed blastocysts were subsequently permeabilized in DPBS-PVA containing 0.5% (*v*/*v*) Triton X-100 and 0.1% sodium citrate (*w*/*v*) for 30 min at room temperature. Then, the samples were incubated in TUNEL reaction medium for 1 h at 38.5 °C in the dark, and nuclear DNA was counterstained with 5 μg/mL Hoechst 33342. Finally, the blastocysts were mounted onto glass slides with Fluoromount ^TM^ aqueous mounting medium, and the fluorescence signal from each slide was analyzed under an Olympus IX83 inverted fluorescence microscope.

### 2.9. Blastocyst Outgrowth Assay

The blastocyst outgrowth assay was performed as described previously [32]. Briefly, zona pellucida (ZP) were removed from blastocysts using Tyrode’s solution. The ZP-free embryos were then cultured in Dulbecco’s modified Eagle medium (DMEM) containing 10% FBS at 38.5 °C in 5% CO_2_. Images of outgrowing embryos were photographed daily using an inverted microscope and the area of trophoblastic outgrowth was measured on 14 dpf using ImageJ software.

### 2.10. Statistical Analyses

The experiments were replicated at least three times in each group. Statistical analyses were performed using the SPSS Statistics Version 25.0 software (IBM Corp., NY, USA). All percentage data of survival rate and developmental rate were subjected to arcsine transformation and analyzed by one-way analysis of variance (ANOVA) followed by Duncan’s test. The remaining data among the experimental groups were analyzed by Student’s t-test. The data are presented as the mean ± SEM. A *p* value < 0.05 was considered statistically significant.

## 3. Results

### 3.1. Effects of NAC Supplementation in IVM Medium on the Expression of NFKB1 Gene in Bovine Oocytes

**Figure 1:**
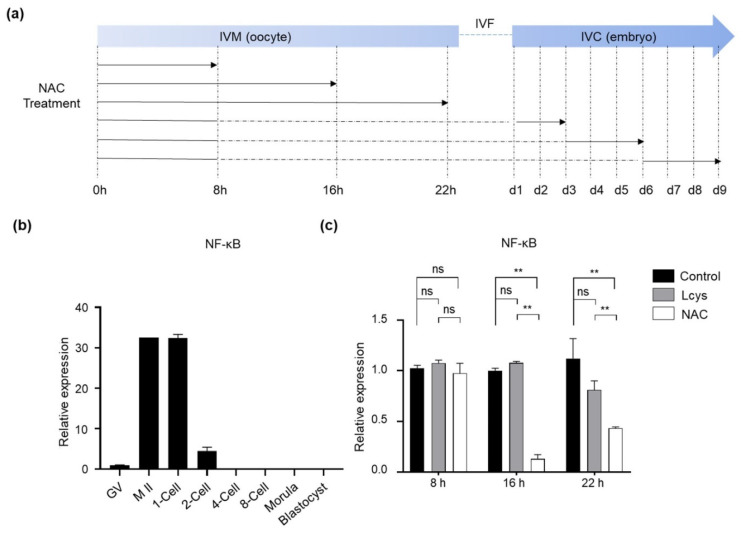
The effects of NAC supplementation on the expression of *NFKB1* gene in bovine oocytes and embryos. (**a**) Schematic of NAC supplementation timeline. Solid lines represent the period that oocytes or embryos were cultured in medium with NAC, while dashed lines represent the period without NAC. (**b**) *NFKB1* mRNA expression levels in oocytes and IVF-derived embryos cultured under basic medium (control). (**c**) Time-dependent inhibitory effects of NAC supplementation during IVM on *NFKB1* expression in oocytes. NAC, N-acetyl-L-cysteine; Lcys, L-cysteine; IVM, in vitro maturation; IVF, in vitro fertilization; IVC, in vitro culture; *NFKB1*, nuclear factor kappa B subunit 1; GV, germinal vesicle; M II, metaphase II. Values are means ± SEM calculated from three replicates; ns, no significant difference; **, *p* < 0.01.

NAC has demonstrated the potential to inhibit NF-κB activation, which is essential for oocyte maturation and early embryo development [33]. In order to avoid the inhibitory effects of NAC supplementation on the pathway, we studied the expression profile of the *NFKB1* gene from immature oocytes to blastocysts in order to define the supplementary time windows (Figure 1a). The results indicate that the *NFKB1* mRNA increased during the stages of oocyte maturity, from the germinal vesicle stage (GV) to the meiosis II (M II) stage, and gradually declined after fertilization; it was almost undetectable after the 2-cell stage (Figure 1b). Incubating the COCs in the presence of 1.0 mM NAC for longer than 8 h significantly reduced the expression level of *NFKB1* mRNA compared to the L-cysteine and control group (Figure 1c). Therefore, the COCs were selected to be incubated with NAC only for the first 8 h in IVM in subsequent experiments.

### 3.2. Effects of NAC Supplementation in IVM Medium on Oocyte Maturation and Embryo Development

COCs were incubated with increasing concentrations of NAC (0.1 mM, 1 mM and 10 mM) and 0.6 mM of L-cysteine, respectively; then, the polar body extrusion rates were examined after 22 h of IVM (Table 2). The results show that treatment with concentrations as high as 10 mM of NAC during the IVM period is detrimental to the growth of oocytes, while reducing the concentration to 0.1 mM failed to promote oocyte maturation and subsequent embryo development. One millimolar of NAC significantly increased the maturation rate of oocytes comparable to 0.6 mM L-cysteine (83.06% vs. 82.76%), both of which are definitely higher than the control group. Although the supplementation of 1.0 mM NAC in IVM medium did not impact cleavage rates compared to the control group, it increased the proportion of top-quality 2-cell embryos (92.93% vs. 88.30%) (Supplementary Figure S2), blast formation rate (41.69% vs. 37.15%) and hatching rate (60.65% vs. 54.77%). On the contrary, L-cysteine only improved the maturation rate and cleavage rate, but these beneficial effects did not continue up to the blastocyst stage (Table 2). These findings demonstrate that NAC supplementation in IVM medium could effectively improve oocyte maturation and enhance the developmental ability of embryos over the 2-cell stage than L-cysteine.

### 3.3. Effects of NAC Supplementation in IVM Medium on Bovine Oocyte Quality

**Figure 2:**
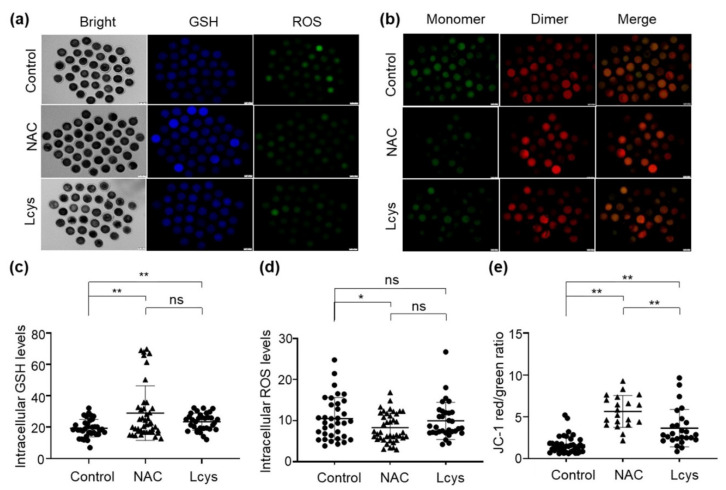
Supplementation of NAC increases the quality of in vitro mature oocytes. Intracellular GSH, ROS levels (**a**) and mitochondrial membrane potential (**b**) of oocytes incubated in medium with or without NAC (1 mM) and L-cysteine (0.6 mM). Images were analyzed by an inverted two-deck microscopy system (IX83, Olympus, Japan) with identical fluorescence parameters. Normal mitochondrial membrane potential is visualized in red with JC-1 dimers and depolarized membrane potential is shown in green with JC-1 monomers. Fluorescence signal of GSH (**c**), ROS (**d**) and mitochondrial membrane potential (**e**) in the images were quantified by using the ImageJ software. Scale bars = 100 μm. NAC, N-acetyl-L-cysteine; Lcys, L-cysteine; GSH, reduced glutathione; ROS, reactive oxygen species. Values are means ± SEM calculated from three replicates; ns, no significant difference; *, *p* < 0.05; **, *p* < 0.01.

The levels of ROS and GSH in matured oocytes are important indicators of oocyte quality. We found the GSH levels in the oocytes treated with 1 mM NAC or L-cysteine were obviously increased (Figure 2a,c), while ROS levels were significantly decreased (Figure 2a,d) compared to the oocytes in the untreated group (*p* < 0.05). To determine the mitochondrial membrane potential (ΔΨm), we calculated the green/red fluorescence intensity ratio in each oocyte. As shown in Figure 2b, oocytes from the control group showed a relatively low ratio, while the oocytes treated with NAC or L-cysteine exhibited significantly higher ratios; there is a significant difference between the NAC- and L-cysteine-treated groups (Figure 2b,e). These results suggest the significant efficacy of NAC supplementation regarding the antioxidant ability and maintenance of mitochondrial function during IVM.

### 3.4. Effects of NAC Supplementation in IVM Medium on Bovine Cumulus Cells Quality

**Figure 3:**
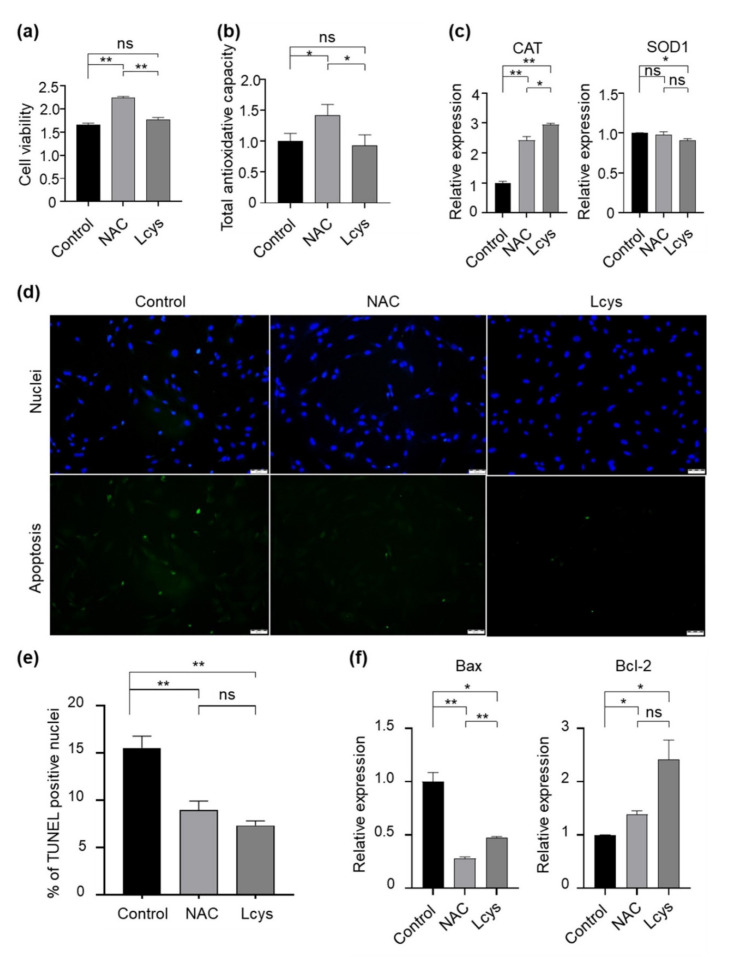
Supplementation of NAC and L-cysteine improves the quality of cumulus cells peeled from oocytes matured in vitro. (**a**) Cell viability was determined with an MTT assay. (**b**) Total antioxidant capacity of cellular content (TAC) was analyzed by an ELISA kit following the manufacturer’s instructions. (**c**) The expression levels of antioxidase genes (*CAT* and *SOD1*) were detected by real-time qPCR and normalized with *GAPDH*. (**d**,**e**) Apoptotic cells were detected by the TUNEL assay. (**f**) The expression levels of apoptosis-related genes (*Bax*/*Bcl-2*) were detected by real-time qPCR and normalized with *GAPDH*. Scale bars = 100 μm. NAC, N-acetyl-L-cysteine; Lcys, L-cysteine. Values are means ± SEM calculated from three replicates; ns, no significant difference; *, *p* < 0.05; **, *p* < 0.01.

Cumulus cells surrounding the oocytes play important roles for the developmental competence of the oocytes. Therefore, we evaluated the quality of the cumulus cells during the IVM period in the presence of NAC or L-cysteine. We did not find statistical differences in cell viability (Figure 3a) and total antioxidant capability (Figure 3b) in the cellular content of cumulus cells between control and L-cysteine-treated groups, whereas in NAC-treated groups, they were dramatically improved. In addition, NAC maintained the expression of the *SOD1* gene to a level comparable to the control group, and presented an obviously higher level of *CAT* gene expression in cumulus cells (Figure 3c). Moreover, we found that among each experimental group, NAC supplementation significantly reduced the proportion of apoptotic cells (Figure 3d), downregulated the expression of the pro-apoptotic *Bax* gene and upregulated the expression of the anti-apoptotic *Bcl-2* gene (Figure 3e). These results indicate that, although L-cysteine has similar protective effects on cumulus cells to NAC in several aspects, NAC has obvious advantages in improving the quality of cumulus cells through maintaining cell viability and antioxidant effects.

### 3.5. Effects of NAC Supplementation in IVC Medium on Bovine Embryo Development

To define the appropriate time window in which NAC promotes embryo development, we restricted the duration of NAC treatment to 0–3 dpf, 3–6 dpf and 6–9 dpf (Figure 1a). As shown in Table 3, a reduced cleavage rate (81.46% vs. 90.25%) and hatching rate (57.20% vs. 58.44%) were observed in the presence of NAC during 0–3 dpf compared to the control group (Table 3). When the presumptive zygotes were incubated in the presence of NAC during 3–6 dpf, both the blastocyst formation rate (49.72% vs. 38.20%) and hatching rate (65.85% vs. 58.44%) were remarkably increased compared to in 0–3 dpf-treated specimens and the control group. Although the treatment with NAC during 6–9 dpf also improved the blastocyst formation rate compared to the control group (48.06% vs. 38.20%), this beneficial effect did not extend to the hatching period (Table 3). These findings suggested that treatment with NAC from 3 dpf is conducive to the developmental competence of bovine embryos.

### 3.6. Effects of NAC Supplementation in IVC Medium on Bovine Embryo Quality

**Figure 4:**
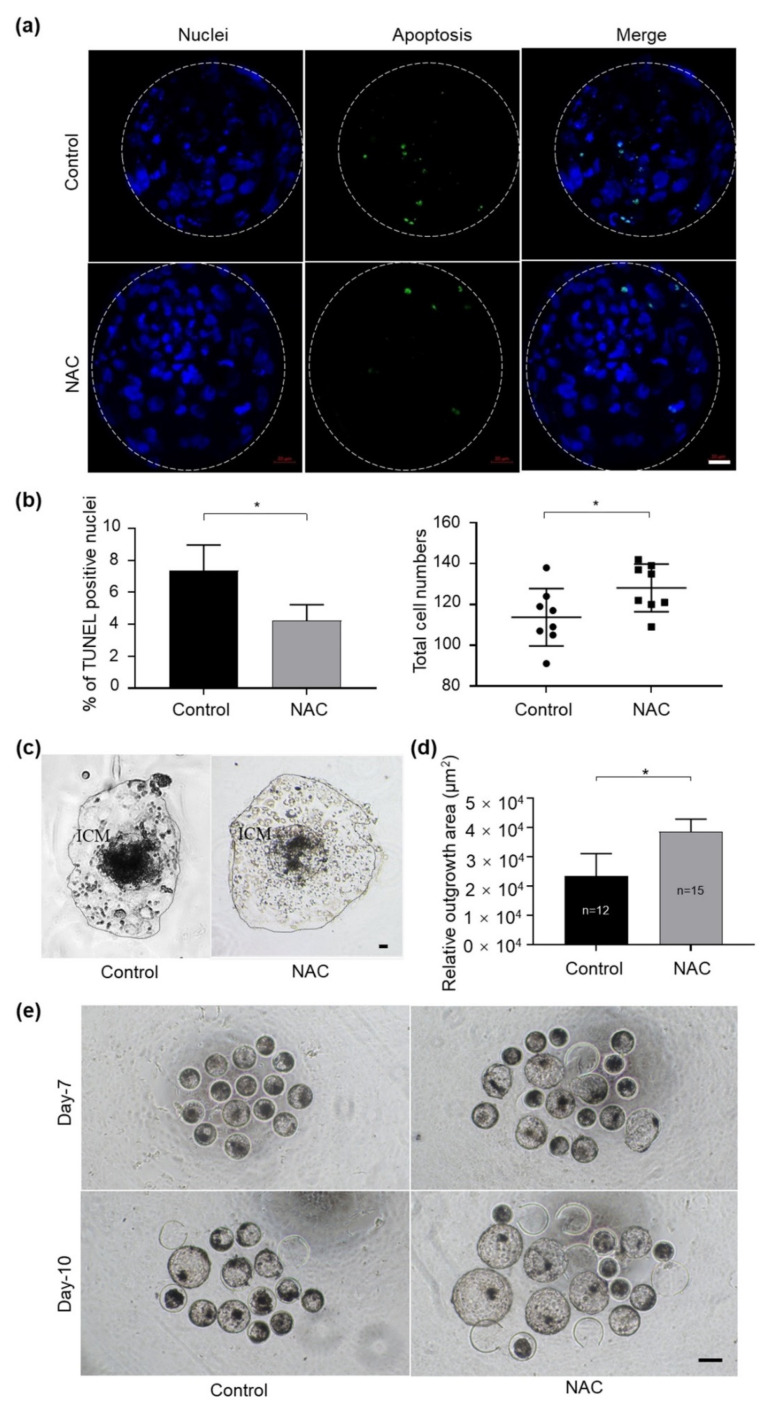
Supplementation of NAC to the culture medium in bovine embryo development. (**a**) Apoptotic cells in blastocysts treated with 1 mM NAC during 3–6 dpf were stained in a TUNEL assay. Nuclear DNA is shown in blue; apoptotic blastomeres are shown in green. Scale bars = 100 μm. (**b**) The apoptotic rate and total cell number were quantified from the fluorescent images with ImageJ software. (**c**) Representative images of the morphology of blastocyst outgrowth on 14 dpf. (**d**) The trophoblastic spreading area was quantified using the ImageJ software. (**e**) Photos taken on 7 dpf and 10 dpf in the presence of NAC during culture. NAC, N-acetyl-L-cysteine; dpf, day post fertilization; ICM, inner cell mass. Values are means ± SEM calculated from three replicates; *, *p* < 0.05.

We further evaluated whether NAC treatment during 3–6 dpf of culture improved the quality of IVF embryos. The results revealed that blastocysts derived from the NAC-treated group exhibited significantly lower TUNEL-positive nuclei per embryo and higher total cell numbers (Figure 4a,b). In addition, NAC supplementation obviously improved the implantation potential of IVF-derived blastocysts compared with those of the respective control by measuring the trophoblastic spreading area using an outgrowth assay (Figure 4c,d). In particular, we noticed that, when the culture medium was supplemented with NAC, more embryos hatched from the zona pellucida on 7 dpf and on 10 dpf compared with the control group, respectively (Figure 4e). These data suggest that NAC supplementation could protect embryos by preventing the blastomere from apoptosis and that it leads to faster embryonic development compared to embryos generated in the control group.

## 4. Discussion

The precise regulation of cellular redox homeostasis is critical for early embryonic development. The oxygen tension in the female oviduct is less than 8%, and in the uterus, it is as low as 3–5% [35]. Thus, the development of preimplantation embryos in vivo takes place under hypoxic conditions, and even anaerobic conditions. However, in vitro culture for gametes and embryos has been performed in atmospheric O_2_ (approximately 21%), supplemented with 5% CO_2_ for decades for various reasons, such as reducing costs [36]. This difference in growing environment between in vivo and in vitro induces oxidative stress or imbalance between oxidants and antioxidants [37], which leads to impaired early development and embryonic fragmentation [35]. The glutathione redox system is deeply involved in embryogenesis to protect pre-implanted embryos from the adverse effects of superoxide such as high levels of ROS in reproductive tract fluid [38]. The GSH level in bovine embryos is highly associated with viability after cryopreservation and early development [12]. The increase in GSH synthesis during oocyte maturation will ensure a sufficient amount of GSH in the embryos against ROS until the blastocyst stage [39]. In the present study, we observed that supplementation of NAC, as well as L-cysteine, in IVM medium can significantly increase the levels of GSH, reduce the levels of ROS (Figure 2), and promote the development of oocytes and subsequent embryos as expected (Table 2). 

Although NAC is more soluble and stable than L-cysteine (Supplementary Figure S1a) and has been used to increase intracellular GSH and alleviate oxidative stress in mitotic cells [40,41,42], its inhibitory effect on the NF-κB signaling pathway is an obstacle for application in oocyte and embryo culture [33]. The NF-κB signaling pathway plays a pivotal role in morphogenesis and embryonic development. The dysregulation of the NF-κB signaling pathway is related to the senescence of mouse [43] and bovine oocytes [44,45]. During spermatogenesis in mice, NF-κB activation occurs in the pachytene and triggers the transcription of related genes in testes during the subsequent differentiation steps [46]. In mouse embryonic development, NF-κB activation is only observed in the early 1-cell stage, which suggests that it may be involved in the formation of 1-cell zygotes from M II-arrested oocytes [33]. Generally, more than 90% of the newly retrieved bovine oocytes from abattoir-derived ovaries are in the GV phase, and nearly 40% of oocytes passed GVBD and entered the metaphase I phase (M I) after 6 h IVM. M II oocytes began to appear gradually from 12 h of IVM [47]. Therefore, accordingly, we set different window periods to study the relationship between NAC and NF-κB pathway-related genes (Figure 1). As we expected, the mRNA of *NFKB1*, coding a subunit of the NF-κB protein complex, was detected in bovine M II oocytes, then dramatically downregulated in 2-cell embryos during preimplantation development (Figure 1b). Continuously treating oocytes with NAC for 22 h significantly suppressed the expression of *NFKB1* (Figure 1c), and this inhibitory effect was relieved when NAC was withdrawn after 8 h of treatment (Figure 1a,c). These findings suggest that culturing oocytes in IVM medium in the presence of NAC for the first 8 h will be beneficial without obvious side effects.

A previous study reported that the concentration of NAC needs to be higher than 1.0 mM to effectively remove ROS [21], and the most commonly used concentrations in cell culture are in the range of 1–10 (or even 20) mM. Clinical studies have shown that the plasma concentration of NAC actually ranges from 300 to 900 mg/L, which is equivalent to 1.8–5.5 mM [48]. When the concentration of NAC achieved in plasma is too low, the deacetylation of NAC inside the erythrocyte will be too slow to provide the L-cysteine required to increase the rate of GSH synthesis [49]. Additionally, our preliminary study showed that supplementation of NAC above 10 mM reduced the pH of the medium and was hard to equilibrate to the physiologically required 7.2 (Supplementary Figure S1b). Therefore, in the present study, we tested the concentration of NAC from 0.1 to 10 mM. Ultimately, we selected the 1.0 mM for follow-up experiments and further revealed that it can effectively enhance the antioxidant capacity of oocytes (Figure 2), cumulus cells (Figure 3) and IVF-derived embryos (Figure 4).

Cumulus cells share close communication with oocytes through gap junctions and paracrine factors to regulate the simultaneous development and maturation of oocytes [49,50]. Cumulus cells also protect oocytes against the damaging effects of oxidative damage [51]. Hence, analyzing the condition of granulosa and cumulus cells is considered among the non-invasive methods for assessing the quality of oocytes [52]. During oocyte maturation, the levels of apoptosis in porcine cumulus cells increased with the extension of incubation time [53]. The apoptotic cells increased approximately 14-fold at 23 h after IVM, compared to those at the beginning of maturation [54]. The present study demonstrates that NAC supplementation in IVM medium obviously prevented apoptosis in cumulus cells, with an improvement of the cell viability, TAC level and antioxidase expression (Figure 3). These results indicate that NAC improved the quality of cumulus cells and predicted high-quality oocytes.

The protective effects of NAC are also observed in bovine embryonic development. We found that embryos treated with NAC from 3 dpf significantly improved the blastocyst formation rate and hatching rate (Table 3). These embryos exhibited fewer apoptotic blastomeres and a higher total cell number (Figure 4), which would attribute to a high level of development potential [32]. The embryonic outgrowth assay is generally used to study trophoblastic invasion during embryo implantation and as an in vitro model to indirectly evaluate the implantation potential of blastocysts [32]. Accordingly, in the present study, the supplementation of NAC during IVC dramatically increased the trophoblastic spreading area compared with the control group (Figure 4c,d). These results further supported our hypothesis that NAC supplementation can improve the development potential of blastocysts. In addition, we noticed that supplementing NAC during IVC shortens the cleavage time and hatching time of the embryo (Figure 4e). This phenomenon has also been reported in some other antioxidants [55], but as for its influence on later embryonic development, further research is needed.

## 5. Conclusions

**Figure 5:**
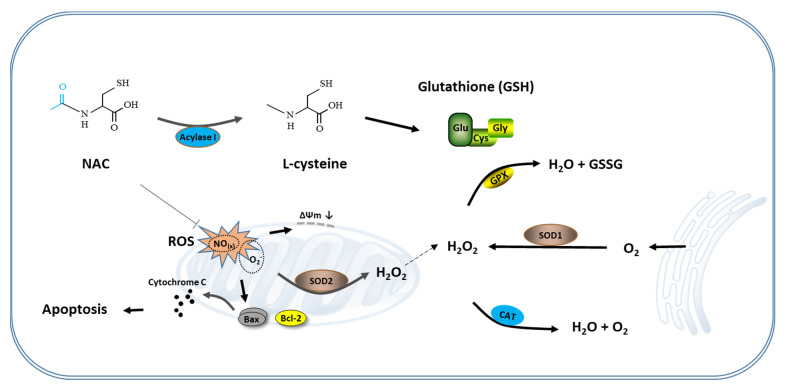
Schematic representation of the antioxidant mechanism of NAC. During the in vitro culture of the cumulus oocyte complex or IVF-derived embryos, excessive ROS produced in the cells lead to mitochondrial-mediated apoptosis. Supplementation of NAC to the culture medium eliminates these ROS and protects cumulus cells and blastomeres from apoptosis by prompting the synthesis of GSH and maintaining mitochondria function, thereby improving the developmental competence of oocytes and IVF-derived embryos. NAC, N-acetyl-L-cysteine.

In conclusion, this study is a systematic study of the effects of NAC on the maturation of oocytes and the development of bovine embryos. Although it is necessary to pay attention to the treatment duration, concentration and the effect on the NF-κB signaling pathway when using NAC in the IVM and IVC process, NAC itself can significantly improve the quality of oocytes and embryos by increasing intracellular GSH synthesis, reducing ROS production, and inhibiting cell apoptosis (Figure 5). Our results suggest that NAC could be used as a preventive antioxidant for in vitro embryo production.

## Figures and Tables

**Table 1:** Primers used in this study.

Gene	Primer Sequences (5′-3′)	Accession Number	Product Size (bp)
Bax	GGCTGGACATTGGACTTCCTTC	NM_173894	112
	TGGTCACTGTCTGCCATGTGG		
Bcl-2	TGGATGACCGAGTACCTGAA	NM_001166486.1	123
	GAGACAGCCAGGAGAAATCAAA		
SOD1	CCACGTCCATCAGTTTGGAGA	NM_174615.2	92
	CTTTTGGCCCACCGTGTTTT		
CAT	CTGGGACCCAACTATCTCCA	NM_001035386.2	148
	GATGCTCGGGAGCACTAAAG		
NFKB1	TGGCGGAATTACCTTCCATAC	DQ464067	110
	CATCACTCTTGCCACAACTTTC		
GAPDH	CCCAGAATATCATCCCTGCT	NM_001034034	185
	CTGCTTCACCACCTTCTTGA		

**Table 2:** Effect of different concentrations of NAC in IVM medium on bovine oocyte and embryo development.

Group	No. (%, ± SEM) of Mature/Retrieved Oocytes	No. of Oocytes Used for IVF	No. (%, ± SEM) of IVF Embryos Developed to			
			2-Cell	Top-Quality 2-Cell ^1^	Blastocyst	Hatched Embryo
IVM medium (Control)	236/299	219	185	163	69	38
	(79.03 ± 0.22) ^b^		(83.56 ± 0.40) ^b^	(88.30 ± 0.65) ^b^	(37.15 ± 1.99) ^b,2^	(54.77 ± 0.14) ^b^
IVM + Lcys (0.6 mM)	182/220	250	231	206	90	51
	(82.76 ± 1.08) ^a^		(91.42 ± 1.59) ^a^	(89.26 ± 1.48) ^b^	(38.97 ± 1.88) ^ab^	(57.22 ± 1.04) ^b^
IVM + NAC (0.1 mM)	178/231	233	192	166	72	40
	(77.10 ± 0.18) ^bc^		(82.74 ± 1.97) ^b^	(86.71 ± 0.97) ^b^	(37.73 ± 0.80) ^ab^	(55.94 ± 1.56) ^b^
IVM + NAC (1.0 mM)	167/201	246	211	195	88	53
	(83.06 ± 1.85) ^a^		(85.86 ± 0.46) ^b^	(92.93 ± 1.38) ^a^	(41.69 ± 0.63) ^a^	(60.65 ± 0.44) ^a^
IVM + NAC (10.0 mM)	188/248	205	160	132	59	12
	(75.69 ± 0.55) ^c^		(78.59 ± 1.03) ^c^	(82.28 ± 1.39) ^c^	(37.19 ± 0.26) ^b^	(19.42 ± 1.49) ^c^

^1^ The embryos are scored following the Istanbul consensus [34]. ^2^ Values with different letters within a column indicate significant differences (*p* < 0.05).

**Table 3:** Time-dependent effects of NAC supplementation during in vitro culture on bovine embryo development.

Oocytes Treated	No. of Zygotes for Culture	No. (%, ± SEM) of Embryos Developed to		
		2-Cell	Blastocyst	Hatched Embryo
Basic CR1aa (Control)	398	361 (90.25 ± 0.26) ^a,1^	137 (38.20 ± 0.43) ^c^	80 (58.44 ± 1.18) ^b^
CR1aa + NAC (0~3 dpf)	346	284 (81.46 ± 1.29) ^b^	127 (44.46 ± 0.42) ^b^	72 (57.20 ± 2.05) ^b^
CR1aa + NAC (3~6 dpf)	295	263 (87.26 ± 1.82) ^a^	130 (49.72 ± 0.72) ^a^	86 (65.85 ± 3.23) ^a^
CR1aa + NAC (6~9 dpf)	342	315 (90.93 ± 0.76) ^a^	151 (48.06 ± 1.84) ^a^	90 (59.44 ± 1.67) ^ab^

**^1^** Values with different letters within a column indicate significant differences (*p* < 0.05).

## Data Availability

Data is contained within the article or Supplementary Material.

## Supplementary Materials

The following are available online at https://www.mdpi.com/article/10.3390/antiox10060860/s1, Figure S1: Comparison of physicochemical properties between NAC and L-cysteine, Figure S2: Morphological analysis of presumptive embryos derived from NAC- or Lcys-treated oocytes.
