# Supplementary material for: N-acetyl-L-cysteine Improves the Developmental Competence of Bovine Oocytes and Embryos Cultured In Vitro by Attenuating Oxidative Damage and Apoptosis
Source: Antioxidants (Basel). 2021 May 27;10(6):860. doi: 10.3390/antiox10060860 (PMC8229896; doi:10.3390/antiox10060860)
Supplement: Supplementary file 1 [file antioxidants-10-00860-s001.zip › antioxidants-1230593-supplementary.pdf]

antioxidants

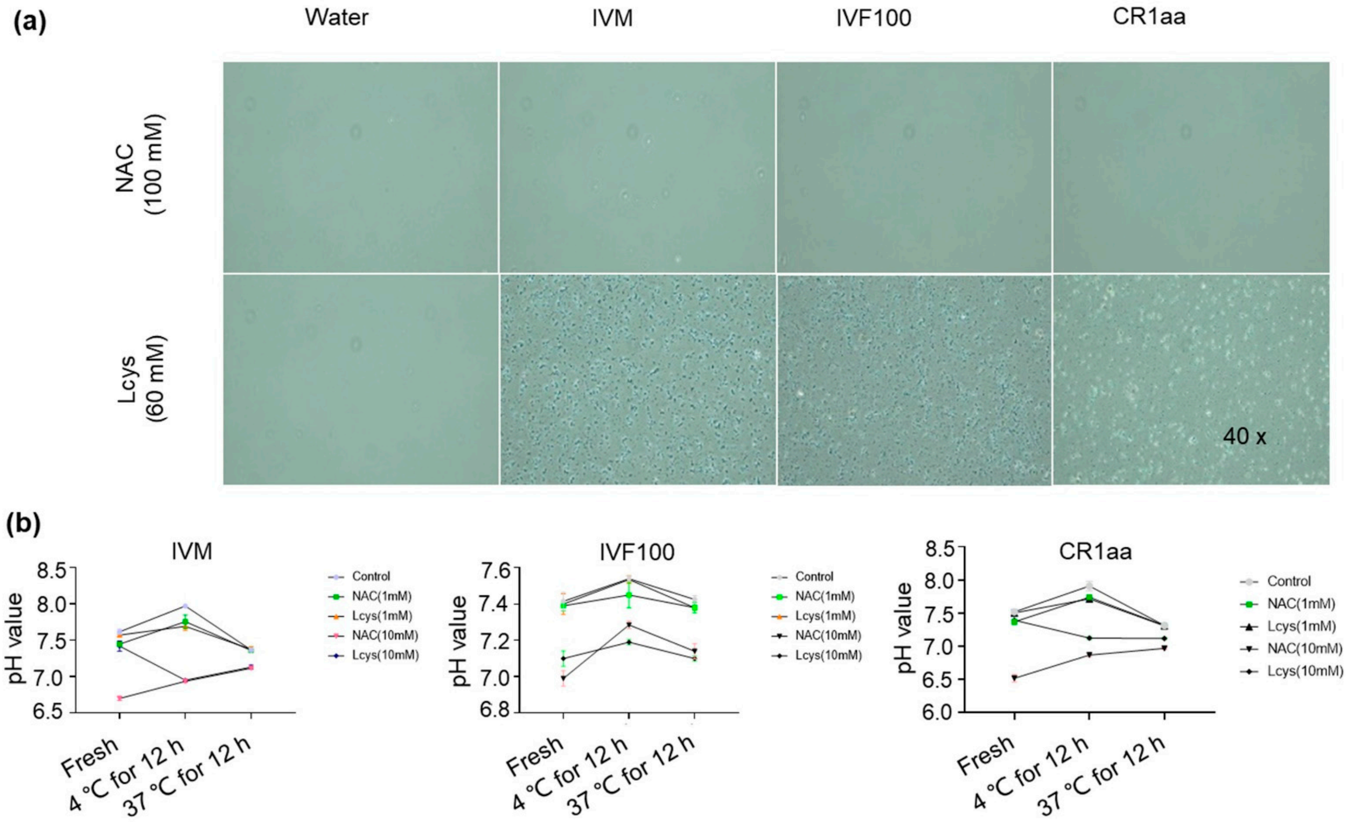

Figure S1. Comparison of physicochemical properties between NAC and L-cysteine. (a) L-cysteine stock solutions (60 mM) stayed clear only when dissolved in water, and presented as white granular precipitate after being dissolved in the other three commonly used culture media (IVM, IVF100, CR1aa) for 1 day at −20 °C. In contrast, NAC did not form any obvious precipitate in all media, even if it was stored for 1 month at −20 °C. (b) Supplementation of NAC above 10 mM reduced the pH of the medium and was hard to equilibrate to the physiologically required 7.2 in the incubator. NAC, N-acetyl-L-cysteine; Lcys, L-cysteine; IVM, in vitro maturation; IVF, in vitro fertilization. CR1aa, Charles Rosenkrans medium with added amino acid.

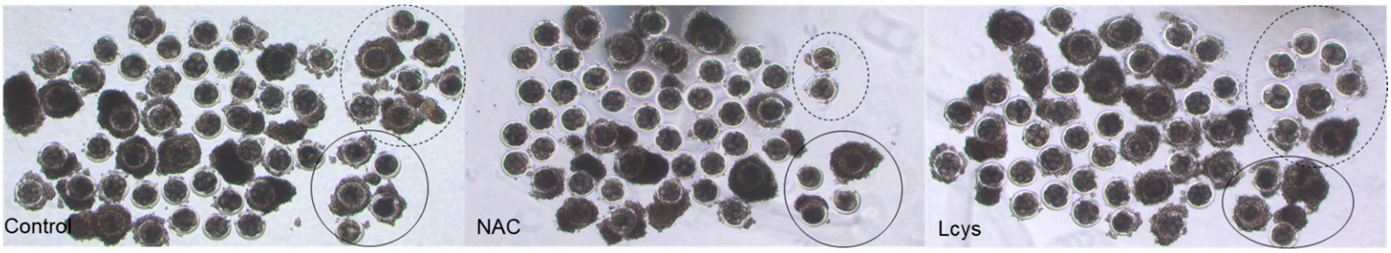

Figure S2. Morphological analysis of presumptive embryos derived from NAC- or Lcys-treated oocytes. Reduced fragmentation and blastomere asymmetry (dashed circles) were observed in the NAC-treated group when compared to the control and Lcys-treated groups. The zygotes that failed to enter the 2-cell stage are shown in open circles. NAC, N-acetyl-L-cysteine; Lcys, L-cysteine.
